# Association of Proteomics Changes with Al-Sensitive Root Zones in Switchgrass

**DOI:** 10.3390/proteomes6020015

**Published:** 2018-03-22

**Authors:** Mahesh Rangu, Zhujia Ye, Sarabjit Bhatti, Suping Zhou, Yong Yang, Tara Fish, Theodore W. Thannhauser

**Affiliations:** 1 Department of Agricultural and Environmental Sciences, College of Agriculture, Tennessee State University, 3500 John Merritt Blvd, Nashville, TN 37209, USA; mahesh.rng@gmail.com (M.R.); zye@my.tnstate.edu (Z.Y.); sbhatti@tnstate.edu (S.B.); 2 R.W. Holley Center for Agriculture and Health, USDA-ARS, Cornell University, Ithaca, NY 14853, USA; yy44@cornell.edu (Y.Y.); tlf26@cornell.edu (T.F.)

**Keywords:** TMT-quantitative proteomics, chromatin remodeling, genome expression reprogramming, protein sumoylation, protein folding, transcription factors, selective gene transcription and translation, physiological stress

## Abstract

In this paper, we report on aluminum (Al)-induced root proteomic changes in switchgrass. After growth in a hydroponic culture system supplemented with 400 μM of Al, plants began to show signs of physiological stress such as a reduction in photosynthetic rate. At this time, the basal 2-cm long root tips were harvested and divided into two segments, each of 1-cm in length, for protein extraction. Al-induced changes in proteomes were identified using tandem mass tags mass spectrometry (TMT-MS)-based quantitative proteomics analysis. A total of 216 proteins (approximately 3.6% of total proteins) showed significant differences between non-Al treated control and treated groups with significant fold change (twice the standard deviation; FDR adjusted *p*-value < 0.05). The apical root tip tissues expressed more dramatic proteome changes (164 significantly changed proteins; 3.9% of total proteins quantified) compared to the elongation/maturation zones (52 significantly changed proteins, 1.1% of total proteins quantified). Significantly changed proteins from the apical 1-cm root apex tissues were clustered into 25 biological pathways; proteins involved in the cell cycle (rotamase FKBP 1 isoforms, and CDC48 protein) were all at a reduced abundance level compared to the non-treated control group. In the root elongation/maturation zone tissues, the identified proteins were placed into 18 pathways, among which proteins involved in secondary metabolism (lignin biosynthesis) were identified. Several STRING protein interaction networks were developed for these Al-induced significantly changed proteins. This study has identified a large number of Al-responsive proteins, including transcription factors, which will be used for exploring new Al tolerance genes and mechanisms. Data are available via ProteomeXchange with identifiers PXD008882 and PXD009125.

## 1. Introduction

Low soil acidity (pH < 5.5) affects about 40% of the world’s arable land [1]. In such soils, the release of excessive amount of Al ions (Al^3+^) has been identified as the major growth-limiting factor that is causing a reduction in crop production [2,3,4]. Susceptible plants develop a stunted root system, resulting in a smaller root to shoot ratio [5,6]. Under the worst scenarios, sensitive plant roots turn brown and die. As most agricultural crop species are susceptible to Al toxicity, the development of tolerant plants is very important for sustaining plant production in affected areas.

Switchgrass (*Panicum virgatum*) is native to the US and Canada. This grass is used as a model crop in bioenergy studies [7,8]. It is known to produce high biomass yield with minimal need for water and fertilizer. It is extremely tolerant to a plethora of environmental stresses that include acid soil (pH < 4.9) that contains high levels of Al^3+^ ions [9]. In our greenhouse studies to screen for Al tolerant plants, we found that switchgrass is highly tolerant to Al stress. In hydroponic culture, these plants were able to survive up to 800 µM Al^3+^ ion (unpublished data, Rangu and Zhou, Tennessee State University, Nashville, TN, USA).

The vertical profile of root tip is divided distally into root-cap, cell division, cell elongation, and maturation zones. The toxic effects of Al occur primarily in the cell division and cell elongation zones. Babourina and Rengel (2009) showed that the primary sites for Al^3+^ entry were at the meristem and distal elongation zones. Al^3+^ uptake also occurs via the cortex and epidermis of the mature root zone [10]. Studies with maize and sorghum have shown that the root distal transition zone is highly sensitive to Al. To overcome this, Al-resistant plants have evolved effective strategies that precisely localize root citrate exudation to the specific site where the greatest root damage can occur [11]. When cells enter the maturation phase, they are no longer very sensitive to Al. Therefore, cells in these distinct root zones respond differently to Al exposure, which could result from the expression of different genes. 

The word “proteome” refers to the total amount of proteins expressed in an organism or in a cell at a certain time [12]. Alterations in the composition of the proteome are one of the major processes used by plants to develop tolerance to suboptimal conditions [13,14,15,16,17,18,19,20]. In previous studies, we demonstrated that under Al stress, tomato plants undergo systemic proteome changes [21]. When compared for Al tolerance/sensitivity, tomato plant roots showed Al toxicity symptoms (brownish color) under 100 µM AlK_2_SO_4_, pH 4.5, whereas the switchgrass roots remained healthy looking (white color with much lateral root growth) at 400 µM AlK_2_SO_4_, pH 4.5. This study was carried out to identify Al-induced proteome changes in switchgrass in order to understand the underlying Al tolerance mechanisms. 

## 2. Materials and Methods

### 2.1. Preparation of Seedling Plants and Al Treatment

Switchgrass seeds were surface sterilized by soaking in a diluted commercial bleach solution (50%) for 10 min followed by 5 rinses with sterile distilled water. Seeds were germinated in sterile deionized water at 29 °C under slow agitation on a rotary shaker for 3 days. Seedlings were transferred into seed cubes to grow to the three-leaf stage when they were transferred into hydroponic tubes. An Al-treatment system was constructed using 6-inch (15.24 cm) diameter PVC pipes [22]. For the control treatments, each tube was filled with 10 L of a modified Magnavaca’s nutrient solution [23]. For the Al-treatments, the tubes were filled with the same solution supplemented with 400 µM AlK_2_SO_4_, pH 4.5 [21]. Solutions were refreshed every ten days. The pH of the solution was measured daily to ensure that it remained below 5.0. Three biological replicates were performed. Each replicate comprised 30 plants. Plants were arranged using a randomized block design.

### 2.2. Physiological Data Collection

Every 7 days, leaf photosynthetic rate, stomatal conductance, and transpiration rate of fully expanded leaves were collected using a LI-COR 6400 Portable-Photosynthesis-System (Li-COR Inc., Lincoln, NE, USA). At harvest, 20 uniform-sized plants in each replicate block were selected to record root lengths, plant heights (from the bottom of the tiller to the top of the latest node), and fresh mass.

### 2.3. Tissue Collection and Preparation of Protein Samples

**Figure 1:**
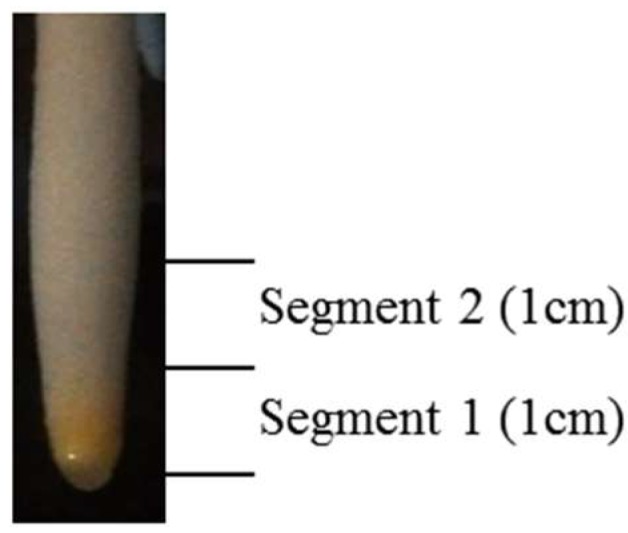
The switchgrass root tissues for proteomics analysis. Segment 1 is the apical root tip tissues that includes root cap, meristematic cell, and some elongating cells, and Segment 2 is the upper 1-cm long region that contains elongation and maturation zone tissues. The two root segments were harvested separately.

Tissues were collected 30 days after the initiation of treatments when plants showed physiological stress symptoms under Al-treated conditions. Two root sections were collected separately: the apical 1-cm cell division that contained root cap and meristem tissues and some elongating cells (Segment 1), and the next apical 1-cm elongation that had some maturation zone tissues (Segment 2) (Figure 1). After dissection from plants, tissues were frozen in liquid nitrogen immediately in a Magenta Box placed in a CryoDewar. When tissues were collected from all the plants in the biological replicate, the tissues were wrapped in a piece of aluminum foil and stored at −80 °C until protein extraction.

For protein extraction, frozen tissues were ground to a fine powder and the powdered samples were washed in a series of solutions: 10% trichloroacetic acid (TCA) in acetone, 80% methanol/0.1 M ammonium acetate, and 80% acetone; then between washes powdered tissue samples were centrifuged at 16,000× *g* for 20 min at 4 °C. Proteins were extracted from pellets using the dense SDS/phenol extraction method [22]. Proteins were precipitated in 0.1 M ammonium acetate in methanol (1:6; *v/v*). Following washes in cold methanol and acetone, the air-dried pellets were solubilized in a buffer containing 500 mM triethylammonium bicarbonate (TEAB), 0.1% SDS, 8 M urea, and 1X protease inhibitors (Sigma, St. Louis, MO, USA) [22]. Urea concentration in protein extracts was reduced to 1 M, TEAB to 100 mM concentration with 50 mM TEAB buffer. Protein concentration was determined by using a Bradford Assay Kit (Bio-Rad, Hercules, CA, USA) [22].

### 2.4. Tandem Mass Tags (TMT) Labeling and Mass Spectrometry Analysis

For each sample, 100 µg of protein was reduced by adding 10 μL of 50 mM Tris (2-carboxyethyl) phosphine hydrochloride (TCEP) followed by incubation at 37 °C for 1 h. Cysteine- groups were blocked at room temperature by adding 6 μL of 200 mM methyl methanethiosulfonate (MMTS) for 10 min. Samples were digested overnight at 32 °C with 2.5 μg of modified sequence grade trypsin (Sigma, St. Louis, MO, USA). The tryptic peptides were labelled with tags (Table 1) using TMT 10-plex Isobaric Label Reagent Set (Thermo Scientific, Rockford, IL, USA) for 1 h at room temperature. The reaction was quenched by the addition of 8 μL of 5% hydroxylamine followed by incubation at room temperature for 15 min. 

One microliter was taken from each of the six labeled samples. They were pooled and cleaned following the ZipTip (Millipore, Billerica, MA, USA) procedure before being used for label check. The remaining labeled samples were combined and subjected to reverse-phase (RP) solid-phase extraction (SPE) procedure to remove SDS and excess tags using cartridges (Sep-Pak C18 cartridge, 1-cm^3^, 50-mg) (Waters; Milford, MA, USA) [22]. Peptides were eluted in 500 µL of 50% (*v/v*) acetonitrile with 0.1% trifluoroacetic acid (TFA) and dried using a CentiVac Concentrator (LabConco, Kansas City, MO, USA). Then, the multiplexed labeled peptide samples were separated, in a high pH first dimension, using an ultra-performance liquid chromatography (UPLC) system (Acquity, Waters) coupled with a robotic fraction collector (Probot; Dionex, Sunnyvale, CA, USA). The separation was achieved on an Acquity UPLC BEH C18 column (1.7 µm, 2.1 mm × 100 mm, Waters, Milford, MA, USA) equilibrated with 20 mM ammonium formate (NH_4_FA) pH 9.5 in water (eluent A) and a 12 min gradient from 10–45% of eluent B [acetonitrile (CAN)/10% 20 mM NH4FA] at a flow rate of 200 µL/min. Forty-eight fractions were collected and concatenated to yield 16-second dimension samples. The concatenated samples were dried under vacuum and reconstituted in 15 µL of 2% acetonitrile with 0.5% formic acid (FA).

Nano-LC-MS/MS analysis was carried out using an Orbitrap Fusion (Thermo-Fisher Scientific, San Jose, CA, USA) mass spectrometer equipped with nano ion source using higher-energy collision dissociation (HCD). The Orbitrap was coupled with an UltiMate3000 RSLCnano (Dionex, Sunnyvale, CA, USA). Each reconstituted fraction (5 µL) was trapped and desalted using PepMap C-18 RP trap column (3 µm, 75 µm (diameter) × 20 mm (length), Dionex) equilibrated with 5% ACN in 0.1% FA at 20 µL/min. Peptides were eluted from the trap column and separated on a PepMap C-18 RP nano column (3 µm, 75 µm × 15 cm) using a 120 min gradient of 5% to 38% ACN in 0.1% FA at 300 nL/min. The Orbitrap Fusion was operated in positive ion mode with nano spray voltage set at 1.6 kV and source temperature at 275 °C. The FT, IT, and quadrupole mass analyzers were calibrated externally. An internal calibration was performed using the background polysiloxane ion signal at *m/z* 445.120025. The instrument was operated in data-dependent acquisition (DDA) mode using the FT mass analyzer to select precursor ions followed by “Top 3 s” data-dependent HCD-MS/MS scans for precursor ions with 2𠄓7 charges/ion above a threshold ion count of 10,000 with normalized collision energy of 37.5%. MS survey scans were carried out at a resolving power of 120,000 (fwhm at *m/z* 200) for a *m/z* range of 400–1600. The AGC and Max IT settings were 3e5 and 50 ms, respectively. MS/MS scans were carried out at 30,000 resolution with the following instrument settings: AGC=1e5, Max IT = 120 ms and the Q isolation window (*m/z*) at 1.6 over the mass range *m/z* 105–2000. Dynamic exclusion parameters were set at 1 within 50 s exclusion duration with ± 10 ppm exclusion mass width. All data were acquired under Xcalibur 3.0 operation software and Orbitrap Fusion Tune 2.0 (Thermo-Fisher Scientific). 

All MS and MS/MS raw spectra from each set of TMT10-plex experiments were processed and searched against the Pvirgatum_v1.1_273_protein database (containing 125439 sequence entries) using Mascot 2.5 (Matrix Science, London, UK). The search settings used were as follows: trypsin with two missed-cleavages; fixed modifications of Methylthio for cysteine, 10-plex TMT modifications on lysine and *N*-terminal amines; and variable modifications of methionine oxidation and deamidation of asparagine and glutamine residues. The peptide mass and fragment mass tolerance values were 10 ppm and 50 mDa, respectively. The TMT10-plex quantification method within Mascot was used to calculate the reporter ratios with a mass tolerance ± 10 ppm without applying isotopic correction factors. Only peptide spectra containing all reporter ions were designated as “quantifiable spectra” and used for peptide/protein quantitation. The mass spectrometry proteomics data have been deposited to the ProteomeXchange Consortium via the PRIDE partner repository with the dataset identifiers PXD009125 and PXD008882 under project title “Association of proteomics changes with Al-sensitive root zones in switchgrass” (http://www.ebi.ac.uk/pride). 

### 2.5. Protein Identification and Quantification, and Statistical Analysis

Proteins with two or more unique peptides were included in the quantitative analysis of proteins. The reporter ion intensity of each peptide was log2 transformed. Then, a t-test (general linear model procedure) of all constituent peptides was performed to obtain a raw p value, and the p values of all proteins were subjected to false discovery rate (FDR) corrections. These two tests give the statistical significance of every protein between the Al-treated and control samples. The log2 ratios of peptides were fitted to a normal distribution [21]. Significantly changed proteins were identified using two standard deviations (±2 SD) of the log2 fold transformed protein ratios and a FDR *p* ≤ 0.05. Protein fold changes were obtained from anti-log conversion of log2 ratios [21,22].

### 2.6. Functional Analysis of Identified Proteins

In the annotated switchgrass database (*Panicum virgatum* v1.1, Phytozome v11.0), each accession was associated with a unigene accession in *Arabidopsis thaliana*. Therefore, the corresponding *A. thaliana* accessions were listed and used to develop protein interaction networks using STRING [24], and functional pathways were developed using MapMan software [25]. Literature searches were conducted to provide additional information to the database-based analysis. 

The SAS version 9.0 software (SAS Inc., Cary, NC, USA) was used to perform the analysis of variance (ANOVA) and the least significant difference (LSD) tests for the physiological data, and *t*-tests and FDR tests for the analysis of quantitative proteomics data.

## 3. Results

### 3.1. Physiological Changes of Switchgrass Plants under Al Treatments

After 30 days of Al treatment, several physiological properties showed significant differences between the Al-treated and non-treated control groups. Leaf photosynthetic rate and transpiration rate of Al-treated plants were lower compared to non-Al treated plants. These physiological changes indicated that the Al-treated plants were under stress conditions. At this time, the experiment was terminated, and root samples for proteomics analysis were harvested (Table 2).

### 3.2. Al-Induced Proteome Changes in Different Zones of Root Tips

#### 3.2.1. Total Root Proteome Changes Induced by Al Treatment

Proteomic analysis identified 6309 proteins from 28,600 unique peptides in Segment 1 (root apex tissues); 4130 proteins were quantified with 2 or more peptides; and 164 (3.9%) were identified as significantly changed proteins (145 at a higher abundance level and 19 at a lower abundance level compared to the non-treated control plants). In Segment 2 (elongation/maturation zones), 7288 proteins were identified from 31,000 unique peptides, and 4636 were quantified with two or more peptides, among which 52 (1.1%) were identified as significantly changed proteins. Seventeen proteins were at a higher abundance level and 35 at a reduced abundance level compared to the non-treated control group (supplementary material Table S1, S2, Table 3). Based on the scale of proteome changes, it is obvious that the apical 1-cm root tip cells underwent a more dynamic change than those of the maturation zone. This concurs with previous reports that the root apex tissues are more sensitive to Al toxicity than those of the maturation zone.

#### 3.2.2. Functional Pathways of Al-induced Significantly Changed Proteins

The Al-induced significantly changed proteins were classified into functional pathways using MapMan. Within each functional pathway, proteins were identified either at a higher abundance level or at a reduced abundance level, compared to the non-treated control group (Table 4). In the Segment 1 tissue in which cell division occurs, proteins (FKBP proteins, CDC 48 protein, supplementary material Table S1) in the cell cycle pathway were identified, but none were found in Segment 2 tissue. In Segment 1, more stress proteins were at a higher abundance level than those at lower abundance level, but more stress proteins were repressed in Segment 2. Proteins in cell organization pathways were induced in both Segment 1 and Segment 2 proteomes. The Al-induced significantly changed proteins were placed in cell wall synthesis and modification, signaling, and metabolic pathways. A large number of these Al-induced significantly changed proteins were placed in the ‘Unknown’ function group.

#### 3.2.3. String Interaction Networks among Al-induced Significantly Changed Proteins

STRING was used to understand the direct and indirect interactions of the Al-induced significantly changed proteins. With this program, we used the corresponding homologous proteins from *A. thaliana* database to predict protein interaction networks [24]. STRING analysis of significantly changed proteins supports the existence of multiple protein-protein interaction networks. Below are a few of the selected networks that might influence Al stress responses in switchgrass roots.

(a) STRING pathway analysis of the Segment 1 (the apical 1-cm root tip tissues) root tip proteins

An interaction network of proteins involved in the assembly of translation machinery was predicted. It was comprised of 11 proteins that cluster together; the three others form a separate branch (supplementary material 3: Figures S2 and S3). The second network contained proteins related to the RNA-splicing mechanism (supplementary material 3: Figure S4); these proteins are involved in RNA capping and RNA alternative splicing. Several transcription factors (TFs) such as basic-leucine zipper (bZIP) TF family protein, nuclear factor Y (NFY), and C2H2-like zinc finger protein were also predicted to form a network. Similarly, a C2H2-type TF [which is sensitive to proton rhizotoxicity (STOP) 1 gene] and homologs were found to play a key role in overcoming the rhizotoxicity from Al and proton (H+) (low pH) in *A. thaliana* and wheat (Triticum aestivum) [26,27]. 

A network of proteins involved in protein folding was identified in the STRING analysis (supplementary material 3: Figure S5). This network was composed of chaperones and heat shock proteins that were at a higher abundance level compared to the non-treated control group. Another network of proteins that was formed in response to this stress consisted of dehydrins, peroxidases, and other stress proteins (supplementary material 3: Figure S6).

(b) STRING analysis of significantly changed proteins in the Segment 2 (root elongation/maturation zones) root proteins

Phenylpropanoid pathway is important for the production of secondary metabolites such as lignin, phenolic acids, and flavonoids [28]. The enrichment of this pathway concurs with the secondary cell wall development of cells in the elongation and maturation zones. Metabolites in this pathway are involved in antioxidant activities, which confer tolerance to plants exposed to toxic metals [29]. A network of phenylpropanoid pathway was observed in the STRING analysis (supplementary material 3: Figure S7a). These proteins were down-regulated. 

Energy deficiency is a general indicator of most types of stress in plants. Plants exposed to Al treatment showed inhibited activities in mitochondrial proteins due to the restriction of respiration, ATP depletion, and reactive oxygen species production [30]. Several significantly changed proteins formed a string network of energy metabolism (supplementary material 3: Figure S7b). These proteins include glyceraldehyde 3-phosphate dehydrogenase-A subunit 2, acyl-CoA-binding protein 6, adenylate kinase 1, and ATPase.

#### 3.2.4. Other Significantly Changed Proteins in Root Tissues

Glutathione S-transferase is an anti-oxidant enzyme that alleviates oxidative stress induced during Al-treatment [31,32,33]. Several peroxidases (Pavir.Bb02758.1.p, treated/control, 0.43, *p* = 0.01 in root apical Segment 1 tissues; Pavir.J00305.1.p, treated/control, 0.65, *p* = 0.001 in segment 2) were at a reduced abundance level compared to the non-treated control group. These proteins have a capacity to overcome oxidative stress induced under Al^3+^ stress conditions [33]. The abundance of rotamase (FKBP 1) proteins that was affecting cell cycle progression (Pavir.Fa00397.1.p, 0.58, *p* < 0.01; Pavir.Fb01976.1.p, 0.51, *p* < 0.01) was reduced in Al-treated root Segment 1 tissues. Xyloglucan endotransglucosylase/hydrolase is a cell wall modification enzyme [34]; its abundance was reduced in Segment 2 tissues (Pavir.J17983.1.p, 0.21, *p* < 0.01). Glutamine synthase (GS) is important for nitrogen assimilation and ammonia re-assimilation, and GS activity was stimulated under excessive Al in wheat [35]. In the Al-treated root Segment 1 tissues, none of the GS proteins were induced; instead, one GS protein abundance was reduced (Pavir.Ib00795.1, 0.56, *p* < 0.01), whereas more than 10 GS proteins did not show significant changes (Pavir.Fa00028.1, Pavir.Fa01410.1, Pavir.J04554.1, Pavir.Da01688.1, Pavir.J11418.1, Pavir.J07933.1, Pavir.J00204.1, Pavir.J12432.1, Pavir.Ia00507.1, Pavir.Ia04860.1, Pavir.Ia02372.1). These results indicate that the responses of GS proteins to Al treatments differ among plants. 

## 4. Discussion

Plants can overcome Al stress by employing tolerance and/or resistance mechanisms. Genes with various functions, encoding transcription factors (TFs) [36,37,38,39], proteins involved in the organic acid exclusion mechanisms (to reduce root uptake of Al^3+^ [40,41,42,43]), and genes that play a role in tolerance/detoxification of internalized Al^3+^ [44,45,46,47,48] have been identified in several plant species. Similarly, based on the annotated functions of Al-induced significantly changed proteins, the following four major mechanisms were proposed for the Al tolerance mechanism in switchgrass roots.

A. Mitigation of Al toxicity through regulation of internalization of Al^3+^ ions and their intercellular sequestration in switchgrass root tips

Release and secretion of organic acids (such as malate and citrate) to form harmless complexes such as the Al-malate complex, thus reducing the concentration of Al^3+^ in rhizosphere, is the most effective Al resistance mechanism in plants [49,50,51]. The release of Al-binding pectinaceous mucilage (alkali-soluble pectin) by border cells (the ‘sloughed off root cap cells’) was found to protect root tips from Al-induced cellular damage [52,53,54]. When Al^3+^ ions are bound with the alkali-soluble pectin, their mobility is greatly reduced, thus reducing their entry into the symplast from the surrounding soil [55].

In this study, we have identified three switchgrass root proteins including Al-activated malate transporter (ALMT1), xyloglucan endotransglucosylase-hydrolase (XTH), and Aluminum Sensitive 3 (ALS3), which could have a role in Al resistance/tolerance mechanisms. The Al-activated malate transporter (ALMT1) gene and homologs have been shown to play a critical role in conferring Al resistance. This occurs as malate forms Al-malate complexes within the root tissue (apoplast and symplast) for internal sequestration from cytosol into apoplast [56,57], or in the rhizosphere, which causes a substantial reduction in the entry of Al into the root tissue [57]. However, the extracellular malate exudation is an energy costly process, and only the apical 3–5 mm of the root releases malate [58]. In the switchgrass, the ALMT1 protein was only identified in root apex but not in the elongation/maturation zone tissues. This observation supports the notion that the Al-induced expression of the ALMT1 is under very tight control in specific root cells as an energy efficient detoxification mechanism. 

The cell wall is one of the primary destinations of internalized Al ions. The primary plant cell walls are composed of cellulose, hemicelluloses, and glycoproteins embedded in a pectic matrix [59]. Studies have shown that the hemicellulose component may impact Al resistance. Lower xyloglucan content is associated with reduced Al-binding capacity in the cell walls of *A. thaliana* [60]. The *A. thaliana* xyloglucan endotransglucosylase-hydrolase 31 (XTH31) has predominately xyloglucan endohydrolase activity in vitro; loss of XTH31 results in a remarkably reduced in vivo xyloglucan endotransglucosylase (XET) action and enhanced Al resistance [34]. The abundance of the switchgrass XTH (Pavir.J17983.1) was reduced 5-fold in Al-treated (ratio of Al treated/control = 0.20) root tip tissues, which suggests that this enzyme may have had a positive impact on Al tolerance following the same mechanism as in *A. thaliana*. More importantly, our finding that the switchgrass XTH protein was regulated under Al stress suggests that these proteins may have an essential role against Al toxicity in both dicots and monocots. 

The ALS3 gene encodes a membrane ABC transporter-like protein. It is localized in the phloem and the root cortex following Al treatment [61]. NAP3 (also known as ABCI17) is a non-intrinsic ABC protein. ALS3 and NAP3 are two genes that function in Al tolerance, as well as the phosphate deficiency condition, which is a common occurrence in Al-enriched acidic soils [62]. ALS3 and NAP3 proteins form an ABC transporter complex, which helps to remove internalized Al away from sensitive tissues such as growing roots to tissues that are less sensitive to the toxic effects of Al [61,62]. The protein abundance of NAP3 (Pavir.Db00270.1) was increased 2-fold in the root-apex, and 1.5-fold in the elongation/maturation zone by Al treatments. The enrichment of NAP3 is in agreement with its function in Al tolerance. However, the ALS3 protein was not quantified with high confidence (it was only identified with one peptide); its role in Al stress cannot be determined in this study.

B. Regulation of cell proliferation and morphological changes in Al-treated roots

Plants shape their organs with a precision demanded by optimal function. Organ shaping requires control over cell wall expansion anisotropy [60]. Swollen root tips and shortened root apex [21] are typical symptoms of Al toxicity [63]. The swollen root tips (or radial swelling) were suggested to be caused by impaired anisotropic growth when grown under non-permissive conditions [63]. In the switchgrass root tips, the tubulin-folding protein (KIS) was at a higher abundance level compared to the non-treated control group (Pavir.Ab03232.1, 2.0-fold). Previous studies showed that KIS mutants produced phenotypes (meiotic defects, impaired cell division, and trichomes bulged and less branched) associated with impaired microtubule function [64]. The switchgrass SPIRAL1-like1 (Pavir.Ib02055.1, 1.43-fold) participates in maintaining the cortical microtubule organization and thus is essential for anisotropic cell growth. A study by Xu (2008) shows that the swollen root tip formation process can be stopped by halting ethylene biosynthesis [65]. In switchgrass, the abundance of a key enzyme for ethylene synthesis, aminocyclopropanecarboxylate oxidase, was reduced (Pavir.Hb01502.1, 0.54-fold). The Al-induced changes in the level of accumulation of these proteins and their role in regulating cell morphology and hormone level may prevent root-tips from becoming misshaped in Al-treated plants.

C. Modulation of the genome expression system

Reprogramming genome expression includes chromatin remodeling, transcription of a selective set of genes, and translation of the encoded proteins. This provides the bases for the global proteome changes described above. A large number of significantly changed proteins were found to be involved in this process. Among the transcription factors/activators, the multiprotein bridging factor 1A (MBF1A) transcriptional coactivator was at a higher abundance level in Al-treated plants compared to control plants (Pavir.Fa01472.1, 1.70-fold); the same protein was also identified in long-term Al-treated tomato roots [21]. BTF3-Basic transcription factor 3 (Pavir.Ia01716.1, 1.97-fold) functions as a key regulator of plant growth and development, as well as in the tolerance to biotic and abiotic stresses [66]. The G-box binding factor 1 (GBF1) is a transcriptional activator (Pavir.Ba03287.1, 1.47-fold) that is involved in the regulatory pathways that activate expression of antioxidant enzymes to control intracellular H_2_O_2_ content [67]. This study of switchgrass root proteomes has identified, for the first time, the association of these TFs with Al stress. Therefore, future studies of these TFs regulatory pathways will lead to the discovery of novel genes playing roles in Al tolerance mechanisms. 

As shown in the STRING-predicted protein-protein interaction networks, proteins with roles in transcript processing were highly enriched in the Al-treated root-tips. These proteins include the pre-mRNA-splicing factor SYF2, which influences constitutive, as well as the 5′ splice site selection [68] (Pavir.Db00194.1, 1.58-fold); the MOS11, which is responsible for transferring mature mRNA from the nucleus to the cytosol (Pavir.Ib01319.1, 1.79-fold); La1, which binds to the 3' poly(U) terminus of nascent RNA polymerase III transcripts, protecting them from exonuclease digestion and facilitating their folding and maturation (Pavir.J16915.1, 1.51-fold); and Embryo defective 3010, which enables selective translation of particular classes of mRNA (Pavir.Ba00390.1, 1.48-fold). The Al-induced changes in the abundance of such proteins may represent a mechanism that ensures that appropriate mRNA species are eventually translated into proteins.

D. Protein post-translational modifications and protection of protein conformation structures

Al^3+^ ions are genotoxic and cause direct damage to DNA [69]. Sumoylation involves small ubiquitin-like modifiers (SUMOs), which are attached to or detached from proteins to modify their function and subcellular localization. SUMOs were found to have a role in counteracting DNA replication stress induced by genotoxic agents [70], thus protecting genome integrity. The Al-induced increase in SUMO1 (Pavir.J23850.1, 1.50-fold) suggests that sumoylation plays a role in prevention of the Al-induced DNA damage in the apical root cell division/elongation zones. 

The internalized Al^3+^ ions induce persistent endoplasmic reticulum oxidative stress, leading to the formation of misfolded proteins [71,72,73]. The accumulation of these misfolded proteins and subsequent aggregation of toxic proteins can cause significant cellular damages. Tolerant organisms use a wide array of mechanisms to maintain protein folding in the correct conformation. For instance, protein disulfide-isomerase 5-2 (PDIL5-2) acts as a protein-folding catalyst that interacts with nascent polypeptides to catalyze the formation, isomerization, and reduction or oxidation of disulfide bonds. The switchgrass PDIL5-2 was at a higher abundance level in Al-treated than the non-Al-treated root apex tissues (Pavir.Ga01807.1, 1.42-fold). Furthermore, the Al-induced chaperones in switchgrass roots are also known to be protective of folded proteins against these stress-induced damages. Together, these proteins could play a key role in relieving Al-induced oxidative stress and protecting protein structural stability and proteome homeostasis, and this can play a significant role in enabling these plants to resist Al toxicity.

## 5. Conclusions

In Al-treated switchgrass roots, a larger number of Al-sensitive proteins were identified in the apical 1-cm root tip tissues compared to elongation/maturation zones. The global proteomic changes paralleled the physiological properties of the cell division zone as the most sensitive part of roots to Al-toxicity. The molecular functions of the Al-induced significantly changed proteins corroborated with the cellular activities of the root tissues. For instance, cell cycle proteins were only identified as Al-sensitive in the root apex tissues containing cell division zones. This proteomics study has identified a number of proteins with reported roles in Al-tolerance/resistance, among which were proteins that have not yet described as Al-sensitive including several transcription factors. Studies in our lab showed that switchgrass can tolerate as high as 800 µM Al^3+^ in conditions in which tomato plants are not viable (unpublished data, Rangu and Zhou, 2017). This indicates that switchgrass is highly tolerant of Al. Further studies will focus on validating the function of these switchgrass proteins and their encoding genes in Al tolerance/resistance. 

## Figures and Tables

**Table 1:** The tandem mass tags (TMT) labeling information of switchgrass protein samples.

Treatment	Segment 1 (Apical Root Tissues)		Segment 2 (Elongation/Maturation Zone)	
	Replicate	Labelling Tag	Replicate	Labelling Tag
Control	Control-1	129N	Control-1	128N
	Control-2	129C	Control-2	126
	Control-3	128N	Control-3	129N
400 µM	400 µM-1	126	400 µM-1	129C
	400 µM-2	127C	400 µM-2	127N
	400 µM-3	131	400 µM-3	128C

**Table 2:** Physiological characteristics of switchgrass plants upon Al treatments.

Physiological Measurements	Control	Al-treated
Photosynthetic rate (μmol CO_2_/m^2^/s)	23.43 ± 0.20^A^	18.07 ± 2.76^B^
Conductance (mol H_2_O/m^2^/s)	0.20 ± 0.01^A^	0.18 ± 0.04^A^
Transpiration rate (mmol H_2_O/m^−2^/s)	5.47 ± 0.77^A^	3.79 ± 0.90^B^
Water use efficiency (WUE) (μmol CO_2_/mmol H_2_O)	4.41 ± 0.23^A^	5.97 ± 2.66^A^

Note: Switchgrass plants were grown in 400 µM Al-treated and non-Al treated conditions. Plants were measured every 7 days after the application of Al-treatment. After 30 days, the photosynthetic and transpiration rate showed significant difference between the Al-treated and non-treated control groups. The fully expanded young leaves were used to record the data. Twenty uniform-sized plants were measured in each replicate experiment. Data represent means and standard deviation (SD) of three biological replicates. Control and treatment data that have same superscript letter are not significantly different (*p* ≤ 0.05). Data analysis was performed using SAS.

**Table 3:** Proteins identified using MS analysis from proteomes of Al-treated root tips.

Types of Protein Data	Segment 1 (Apical 1-cm Root-Tip)	Segment 2 (Elongation/Maturation Zone)
Number of identified proteins	6309	7288
Number of proteins identified with 2 or more peptides and quantified	4130	4636
Number of significantly changed proteins	164 (3.9% of quantified proteins)	52 (1.1% of quantified proteins)

**Table 4:** Distribution Al-induced proteins in the significantly enriched functional pathways.

Functional Pathway	Segment 1 (Apical-1 cm Root-Tip)		Segment 2 (Elongation/Maturation Zone)	
	Higher abundance	Lower abundance	Higher abundance	Lower abundance
Amino acid metabolism	1		1	
Cell cycle		3		
Cell organization	5		4	
Cell vesicle transport	2			
Cell wall synthesis and modification proteins	3		1	1
Development	1	1		1
DNA metabolism	2		1	1
Enzyme families	2	1		5
Lipid metabolism	2			1
Major CHO metabolism	2			1
Mitochondrial electron transport	5		2	
*N*-metabolism		1		
Energy metabolism				5
Nucleotide metabolism	1			
Phyto hormone metabolism	2	1		
Protein degradation	5	3		
Protein synthesis	23	1	2	4
Protein targeting	4	1		
Protein post translational modification	5			
Redox	3		1	
RNA metabolism	14			1
Secondary metabolism	3			1
Signaling	4			3
Stress proteins	10	3	1	7
Transport	3	2	1	
Unknown and others	43	2		7
Total number of proteins	145	19	17	35

Note: Switchgrass proteins were annotated as homologous *A. thaliana* proteins in the MapMan software. These functional pathways were constructed by searching these proteins against *A. thaliana* database in MapMan software.

## Supplementary Materials

The following are available at http://www.mdpi.com/2227-7382/6/2/15/s1, Figure S1: Functional pathway classification of Al-induced significantly changed proteins in (i) apical-1cm root apex Segment 1 (and (ii) elongation/maturation Segment 2 tissues of switchgrass). Figure S2: A STRING network of 11 switchgrass apical root tip tissue proteins involved in translation pathway and these 11 proteins showed a higher abundance level under the Al treatment condition. Figure S3: The STRING network that is involved in post transcriptional modifications that occur in 400 μM Al-treated switchgrass apical root tips of switchgrass. Figure S4: The STRING cluster associated with protein folding functions. Figure S5: A STRING cluster of stress-related proteins that shows a higher abundance level in Al-treated apical 1-cm root tip tissues of switchgrass plants. Figure S6: The STRING cluster of proteins in protein metabolic pathway. Figure S7. The STRING clusters of proteins that shows significantly lower abundance levels in root elongation/maturation zones.
