# Supplementary material for: Association of Proteomics Changes with Al-Sensitive Root Zones in Switchgrass
Source: Proteomes. 2018 Mar 22;6(2):15. doi: 10.3390/proteomes6020015 (PMC6027131; doi:10.3390/proteomes6020015)
Supplement: Supplementary file 1 [file proteomes-06-00015-s001.zip › Supplementary materials/Supplementary materials.pdf]

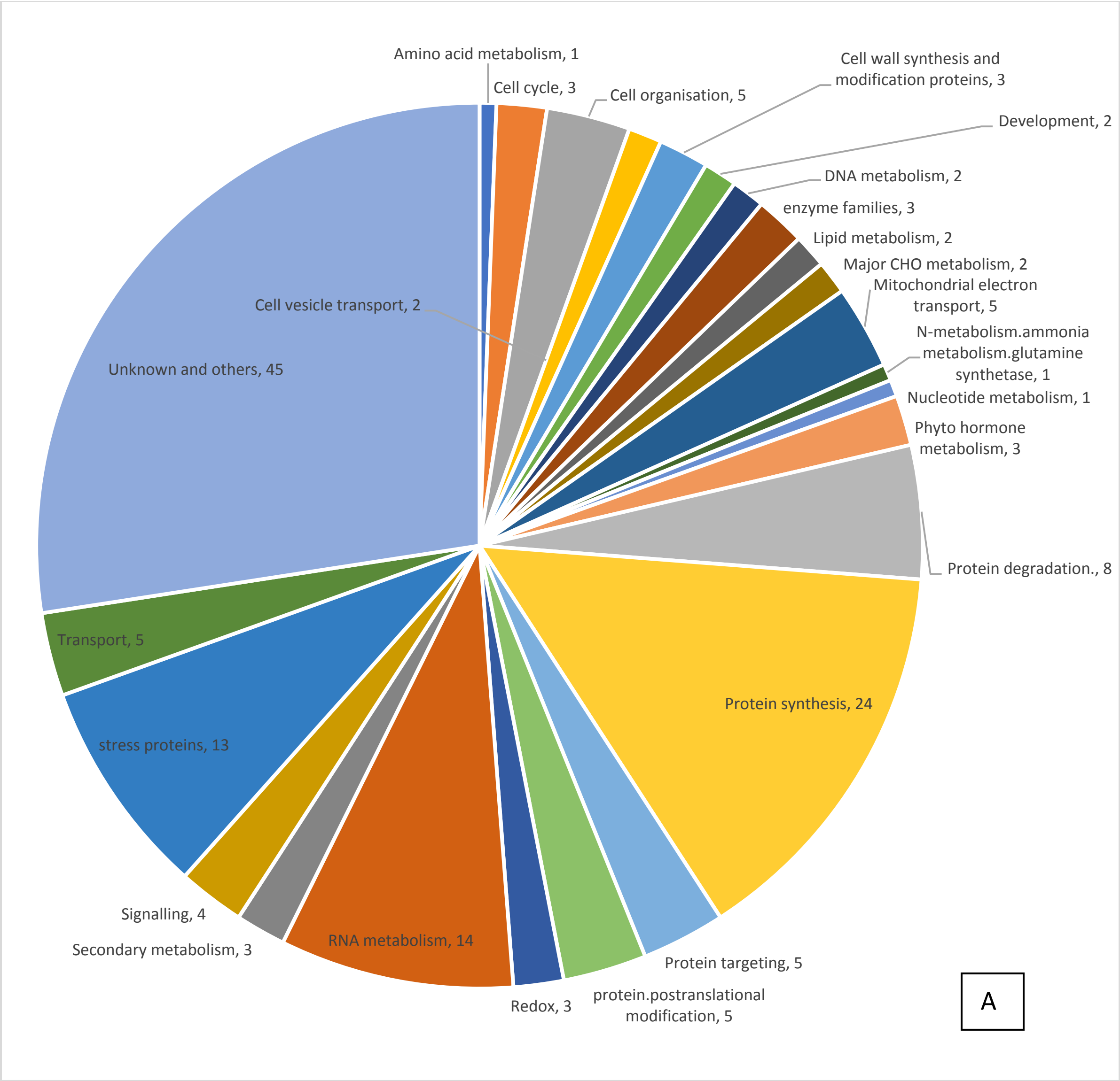
Amino acid metabolism, 1
Cell cycle, 3
Cell organisation, 5
Cell wall synthesis and modification proteins, 3
Development, 2
DNA metabolism, 2
enzyme families, 3
Lipid metabolism, 2
Major CHO metabolism, 2
Mitochondrial electron transport, 5
N-metabolism.ammonia metabolism.glutamine synthetase, 1
Nucleotide metabolism, 1
Phyto hormone metabolism, 3
Protein degradation., 8
Protein synthesis, 24
Protein targeting, 5
protein.postranslational modification, 5
Redox, 3
RNA metabolism, 14
Secondary metabolism, 3
Signalling, 4
stress proteins, 13
Transport, 5
Unknown and others, 45
Cell vesicle transport, 2
A

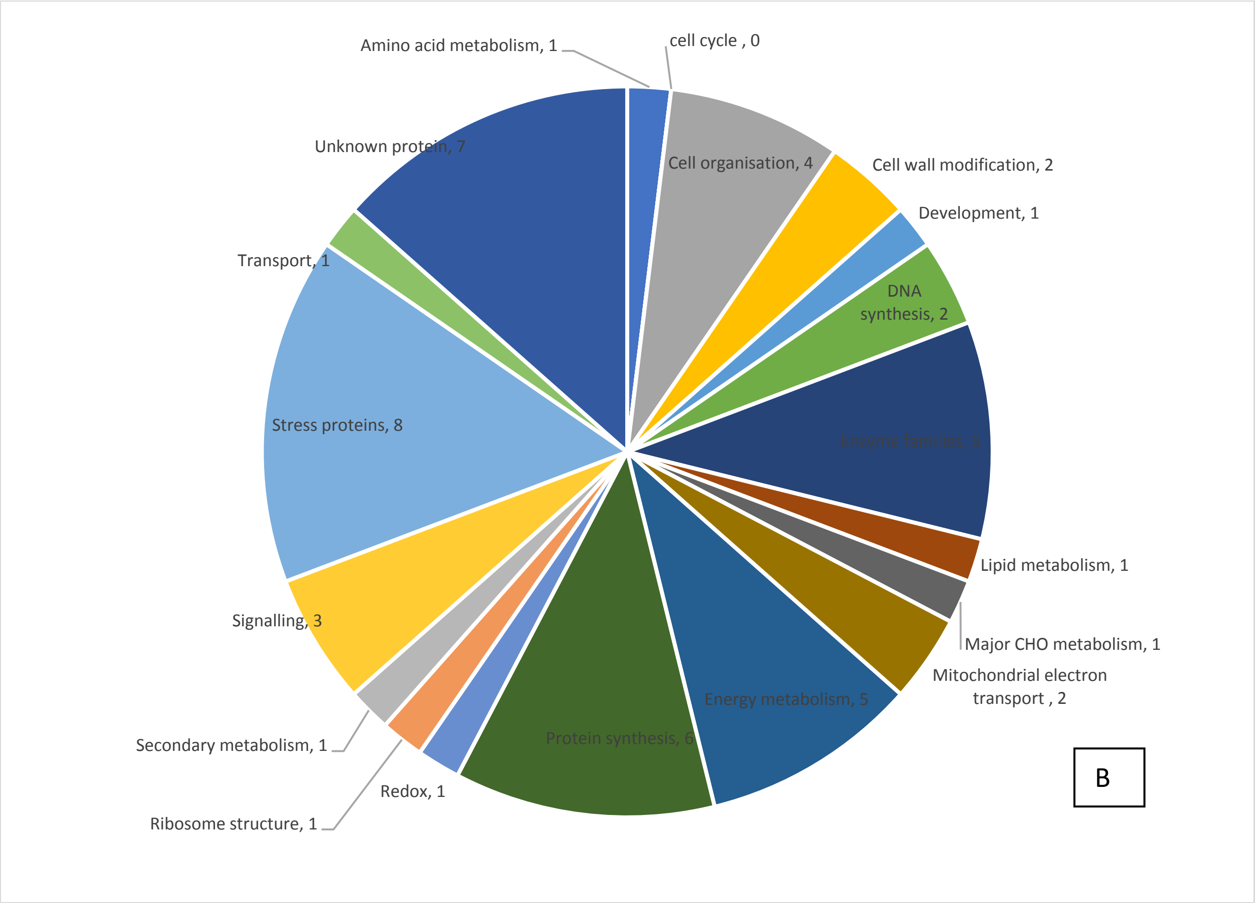

Figure S1: Functional pathway classification of Al-induced significantly changed proteins in (i) apical 1-cm root apex Segment 1 (and (ii) elongation/maturation Segment 2 tissues of switchgrass**.** Each color in the pie chart represents a pathway. The number beside the pathway indicates the number of proteins involved in that pathway. The *Arabidopsis thaliana* protein accessions annotated to these switchgrass proteins were listed in the annotated switchgrass database (*Panicum virgatum* v1.1, Phytozome v11.0). The pathways were developed using *A. thaliana* databases in Mapman.

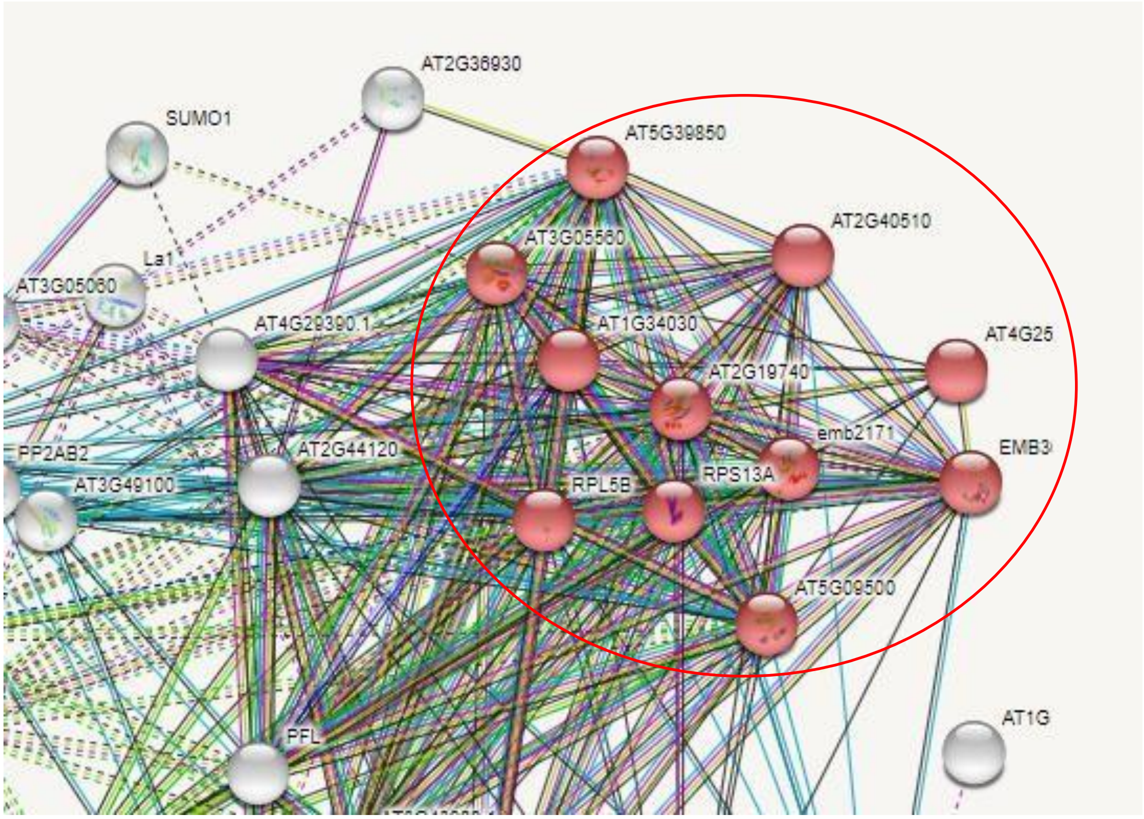

Figure S2. A STRING network of 11 switchgrass apical root tip tissue proteins involved in translation pathway and these 11 proteins showed a higher abundance level under the Al treatment condition. The association network was developed in STRING software using the homologous protein accessions from *Arabidopsis thaliana*.

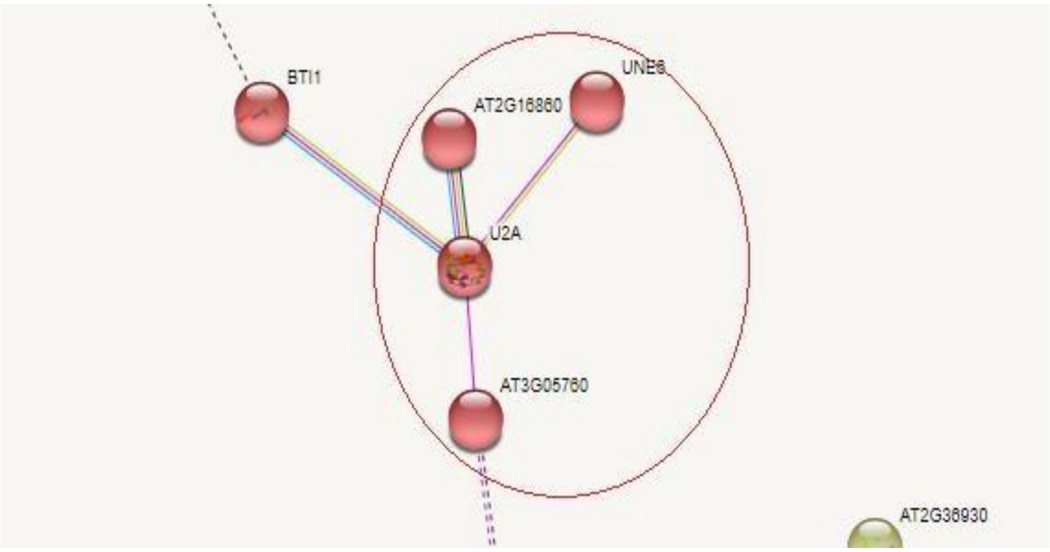

Figure S3. The STRING network involved in post transcriptional modifications occurring in 400 µM Al-treated switchgrass apical root tips of switchgrass. The association network was developed using homologous protein accessions from *Arabidopsis thaliana*.

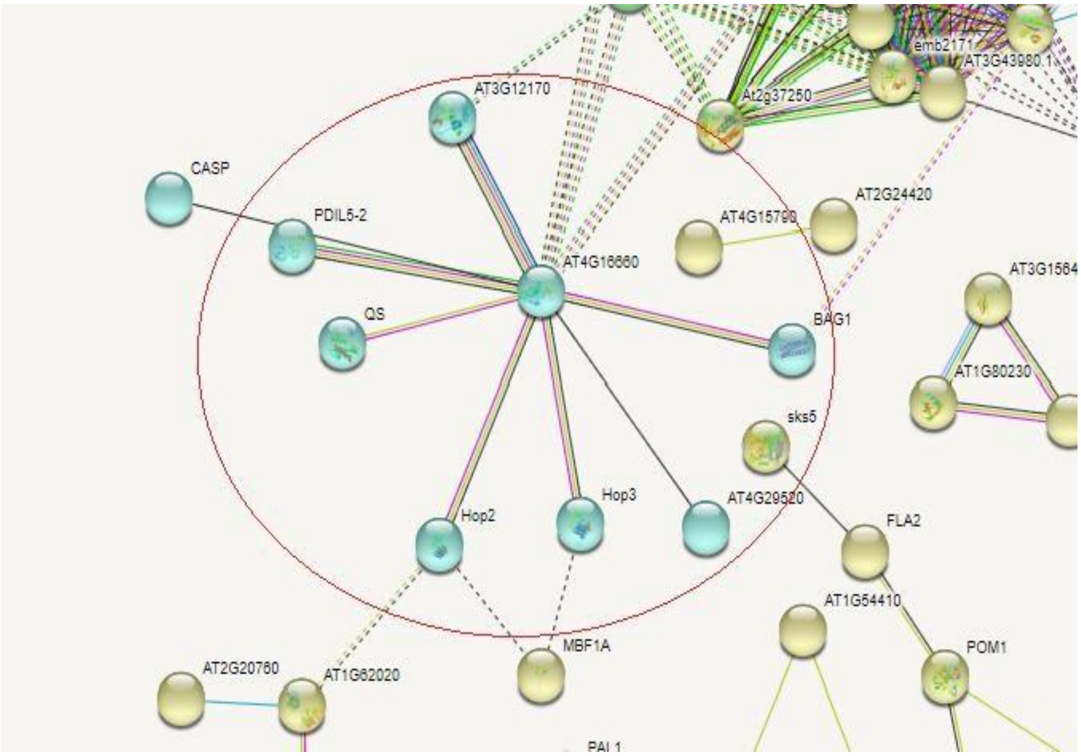

Figure S4. The STRING cluster associated with protein folding functions. These switchgrass proteins had a higher abundance level in Al-treated apical 1-cm root- tip tissues compared to non-treated control plants. The association network was developed using homologous protein accessions from *Arabidopsis thaliana*.

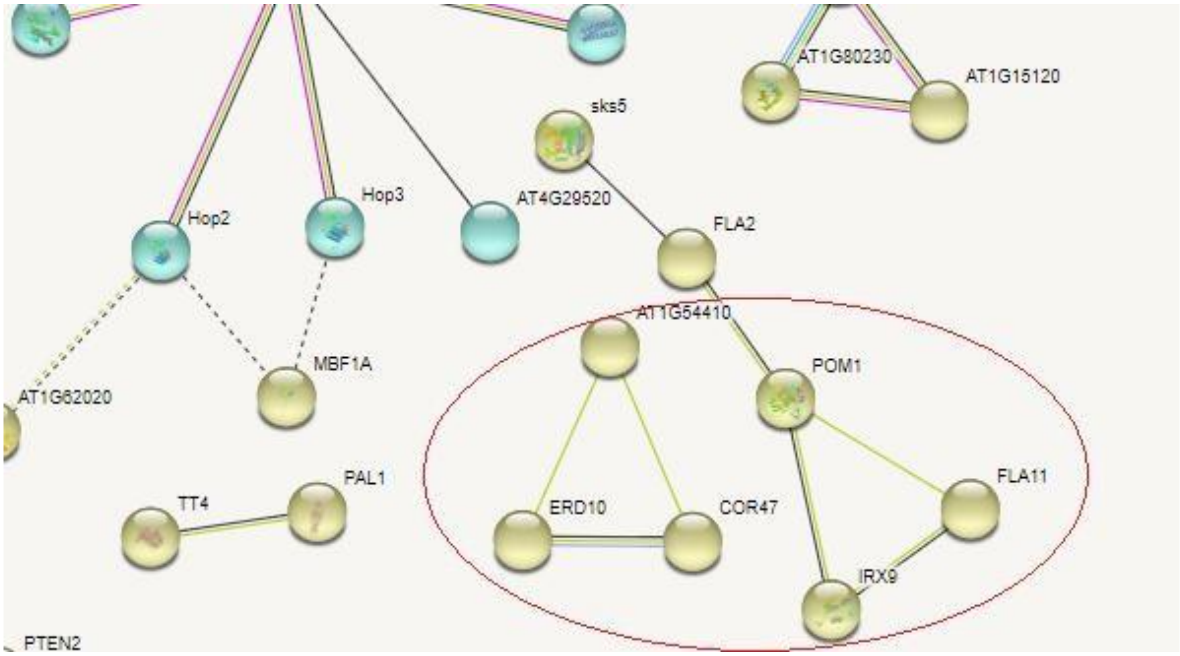

Figure S5. A STRING cluster of stress related proteins showing a higher abundance level in Al-treated apical 1-cm root tip tissues of switchgrass plants. This cluster includes dehydrins and peroxidases and other stress proteins. The association network was developed using homologous protein accessions from *Arabidopsis thaliana*.

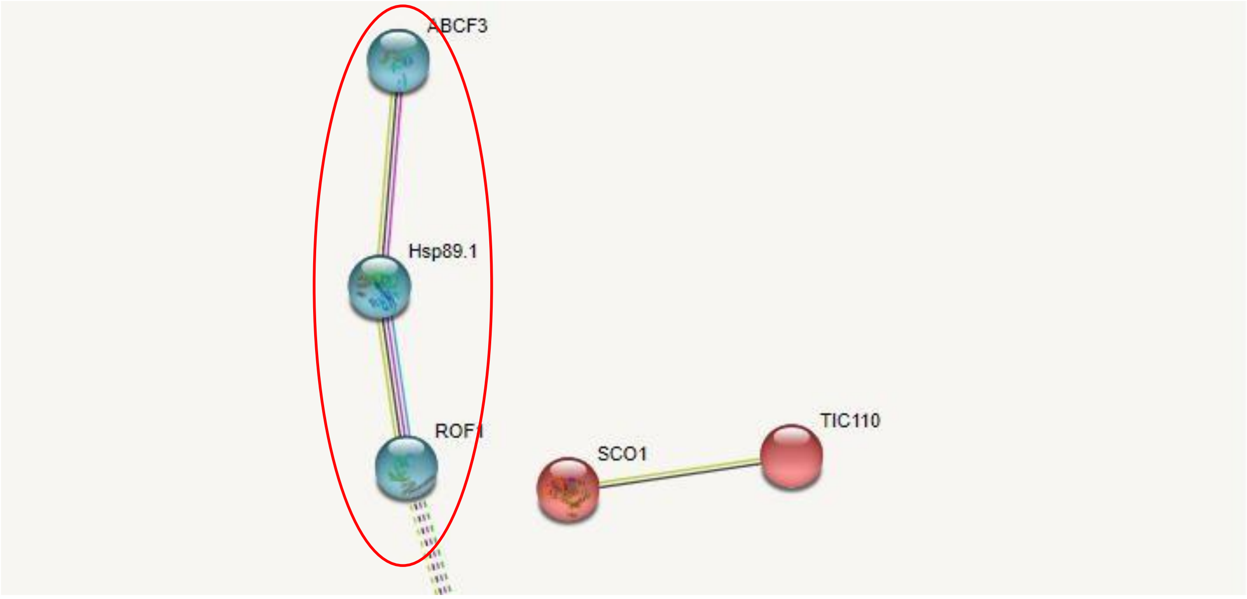

Figure S6. The STRING cluster of proteins in protein metabolic pathway. These proteins were identified at a reduced abundance level in Al-treated apical 1-cm root- tip tissues of switchgrass plants. The association network was developed using homologous protein accessions from *Arabidopsis thaliana*.

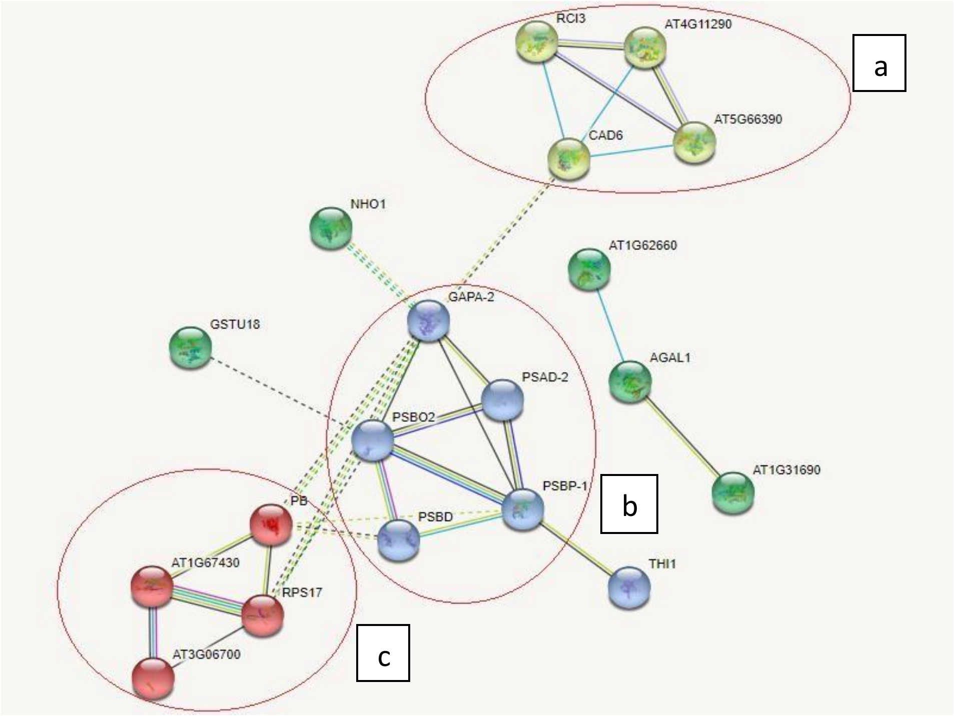

Figure S7. The STRING clusters of proteins showing significantly lower abundance levels in root elongation/maturation zones from the Al-treated to the non-treated switchgrass plants. The association network was developed using homologous protein accessions from *Arabidopsis thaliana*.

a) A cluster of proteins involved in energy-related pathways.
b) A cluster of proteins in the phenylpropanoid pathway.
c) A cluster of proteins involved in protein metabolic pathways.
